# Measuring the Contributions of Perceptual and Attentional Processes in the Complete Composite Face Paradigm

**DOI:** 10.3390/vision7040076

**Published:** 2023-11-17

**Authors:** William Blake Erickson, Dawn R. Weatherford

**Affiliations:** Department of Health and Behavioral Sciences, Texas A&M University-San Antonio, San Antonio, TX 78224, USA; dawn.weatherford@tamusa.edu

**Keywords:** composite face effect, selective attention, holistic processing, divided attention

## Abstract

Theories of holistic face processing vary widely with respect to conceptualizations, paradigms, and stimuli. These divergences have left several theoretical questions unresolved. Namely, the role of attention in face perception is understudied. To rectify this gap in the literature, we combined the complete composite face task (allowing for predictions of multiple theoretical conceptualizations and connecting with a large body of research) with a secondary auditory discrimination task at encoding (to avoid a visual perceptual bottleneck). Participants studied upright, intact faces within a continuous recognition paradigm, which intermixes study and test trials at multiple retention intervals. Within subjects, participants studied faces under full or divided attention. Test faces varied with respect to alignment, congruence, and retention intervals. Overall, we observed the predicted beneficial outcomes of holistic processing (e.g., higher discriminability for Congruent, Aligned faces relative to Congruent, Misaligned faces) that persisted across retention intervals and attention. However, we did not observe the predicted detrimental outcomes of holistic processing (e.g., higher discriminability for Incongruent, Misaligned faces relative to Incongruent, Aligned faces). Because the continuous recognition paradigm exerts particularly strong demands on attention, we interpret these findings through the lens of resource dependency and domain specificity.

## 1. Introduction

Faces are among the most important stimuli that humans encounter. Detecting faces in the environment, encoding them for later recognition, and discriminating among perhaps hundreds of faces are critically important daily tasks. Even though all faces share the same structural configuration of features (i.e., two eyes above a nose and mouth), most people routinely use their facial expertise (and face-selective neurological structures such as occipital face area, fusiform gyrus; [1,2]) to efficiently make judgments about familiarity and identity while carrying out other ongoing tasks. Although these attentional demands are well known, they are not well understood. As a result, this paper seeks to resolve gaps in the literature by measuring the contributions of perceptual and attentional processing to facial encoding and recognition.

### 1.1. Defining and Measuring Holistic Face Processing

Because facial expertise arises out of social and biological necessity, facial researchers have put forth an evolving set of paradigms and theoretical mechanisms to explain this phenomenon. Principally, among the variety of theoretical propositions, one exceptionally prevalent account involves faces occupying a class of visual stimuli that are processed through (at least) two different routes: featural and holistic [3,4,5]. Whereas defining featural processing has received relatively widespread consensus, defining holistic processing is far more varied [6,7]. Holistic processing has been variously defined by encoding of stimuli as (a) global, unified wholes [7,8,9,10], (b) configural elements that respect spatial relations among features (e.g., interocular distance; [11,12,13]), (c) inability to selectively attend to featural information [14,15], and (d) interactions between featural and configural information that operate in parallel [16,17]. Importantly, much of the ambiguity arises from not only differences in definitions of these underlying mechanisms (which are not mutually exclusive), but also in the measures used to operationalize them.

Modern investigations of holistic processing connect back to early work by Tanaka and Farah [7]. The authors investigated whether there is an advantage to recognizing isolated facial features when they are studied in the context of natural, upright faces. Participants in Experiment 1 studied a series of upright faces with all features in a natural, intact configuration or with features scrambled in novel locations on the facial surface. Immediately after each study trial, participants received a two-alternative forced choice test on an individual feature (e.g., a nose). Each choice was presented either in isolation or within the context of a face. An interaction revealed that features from intact faces were better recognized when tested on whole faces; whereas features from scrambled faces were better recognized when tested in isolation.

A follow-up experiment repeated the basic paradigm using an inverted (i.e., upside-down) face comparison group, which the authors believed would sufficiently disrupt holistic encoding while retaining a more natural appearance than scrambled faces. A different interaction manifested such that upright study faces yielded the same pattern as the intact faces from Experiment 1, but the inverted faces showed no difference in whole vs. part recognition. A final study directly compared upright, intact faces to another class of non-face objects with a similarly predictable structure and removable features: houses. Although houses were neither scrambled nor inverted at study, featural recognition (e.g., windows, doors) did not differ within the context of a full house versus in isolation. From these results, a clear pattern emerged. Recognizing individual facial features is tied to the context of face. However, recognizing features of similarly organized non-face objects is not. 

Although this seminal work prompted numerous investigations of holistic processing, it also served as the point from which differing interpretations (in the four main theoretical conceptualizations described above) were put forth using a variety of measures. According to Richler et al. [6], face inversion reveals differences in sensitivity to configuration, whereas scrambling reveals differences in the use of configural and global information. Further, neither approach addresses holistic processing as an inability to attend to featural information and/or interactions between featural and configural information that operate in parallel.

**Figure 1:**
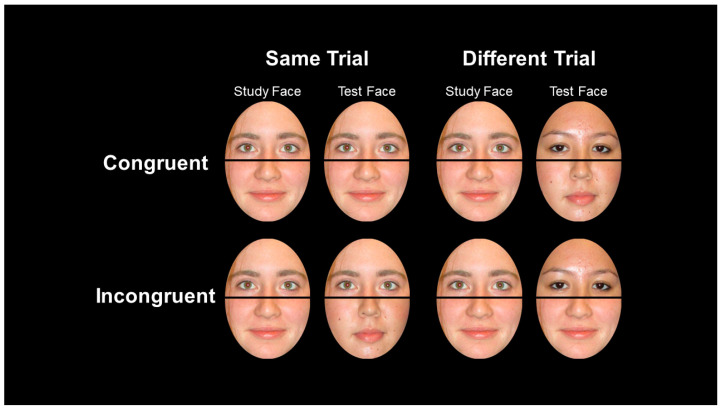
“Complete” design of study and test face combinations across congruent, incongruent, same and different trials. Faces shown are actual stimuli from the current study. All faces in this example are aligned.

**Figure 2:**
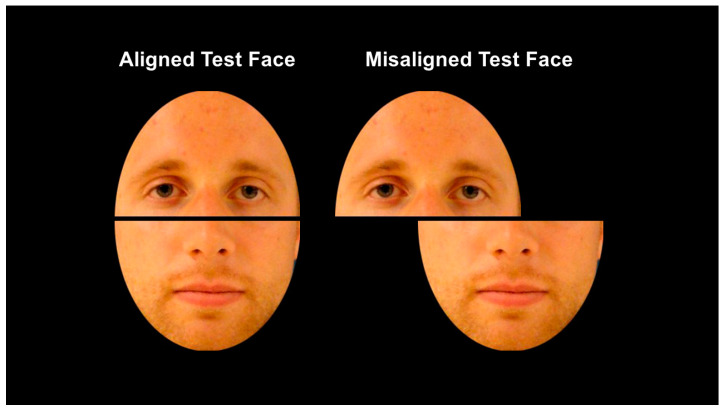
Examples of aligned and misaligned test trial faces.

As a response to this theoretical confusion, Richler & Gauthier [18] developed the composite face paradigm that serves as a measure sensitive enough to vary in response to differences in all four prevailing definitions of holistic processing. This paradigm requires participants to study whole faces that are normally cropped to control for external face shape and hair. Participants then provide a recognition memory judgement to a relevant face half (i.e., top or bottom) in the context of differences in alignment (i.e., either an intact horizontally aligned or misaligned such that the bridge of the nose of the bottom half lines up with the ear of the top half (see, for example, Figure 1 and Figure 2)) and irrelevant face half (i.e., either congruent or incongruent with the correct response to the tested face half). 

In trials where the two congruent halves are aligned, holistic processing facilitates correct responses in line with theoretical positions appealing to the benefits of unified wholes or sensitivity to configural arrangements (see positions a and b above). In trials where two incongruent halves are aligned, holistic processing impedes correct responding in line with failures of selective attention (see position c above). Misalignment disrupts the activation of holistic processing, thereby allowing for comparison of these beneficial and harmful holistic processing effects. 

In early literature using the composite face paradigm (e.g., [19]), a partial design of the task was the version most prominently employed. Although the study trials are the same as the complete composite design, test trials differ. The partial composite paradigm does not manipulate congruence. Further, in some reported experiments, the top half was the focus of the test. Putting aside issues of counterbalancing and participants’ learning to ignore the bottom half of the face and reduce the influence of holistic processing, the partial design does not allow researchers to measure both the positive and negative effects of holistic processing on responding. The full design allows informative analysis of different/new trials as well as the same/old trials. Analyzing results based only on the same test trial trials (e.g., hit rates), to which the partial design is limited, does not permit accounting for response bias as a possible source of variability in the collected data. More importantly, the partial design’s failure to account for response bias may produce opposite conclusions compared to those revealed by the complete design [20]. For these reasons, Richler and Gauthier [18] recommend that the complete design be the paradigm of choice as it minimizes the effects of response bias. 

Subsequent uses of the complete composite face paradigm (and the corresponding composite face illusion) have revealed holistic processing differences as predicted by Richler et al. [6] and Gauthier [21]. Namely, support comes from neurological evidence [22], behavioral evidence in neurotypical populations [23], individual differences in facial recognition ability [24], and face-specific congenital or developmental deficits (e.g., prosopagnosia) [25,26]. Equipped with the appropriate tools, researchers can work towards layering additional manipulations that mirror real-world challenges faced by everyday people, as opposed to adding only partially informative findings to the already confusing theoretical fray. 

### 1.2. The Role of Divided Attention in Face Encoding and Recognition

Although day-to-day face processing happens in a variety of contexts, most laboratory research only examines face encoding and recognition under full attention (FA). If holistic processing is automatic, then it should be impervious to the addition of a secondary task designed to usurp strategic allocation of resources (i.e., divided attention; DA). However, two additional considerations must be taken into account if face processing relies upon at least two routes [3, 4, cf., 5]. First, featural processing is equally, if not more, vulnerable to disruption brought about by DA. As a controlled, resource-demanding route to facial recognition, featural processing should suffer under controlled, resource-demanding DA manipulations. Second, facial memory representations formed at encoding must necessarily be influenced by decisional [27] and attentional [15] systems in order to make accurate recognition judgements [22]. In other words, recognition responses are not an uncontaminated reflection of facial encoding alone. Strategic attentional allocation can influence encoding, retrieval, or both. 

Nevertheless, most research to this point has focused on DA for encoding by using independent study and test blocks. For instance, Reinitz et al. [28] tested memory for line-drawn faces comparing FA to DA brought about by counting dot patterns superimposed on study faces, the number of which participants would later recall during each inter-stimulus interval. Similarly, Palermo and Rhodes [29] instantiated DA by situating the study faces between two flanker faces, and then requiring participants to make same/different identity judgements about those flankers. Both multiple-experimental sequences came to similar conclusions: DA disrupted holistic processing, while leaving featural processing relatively intact. In contrast to this work (and more closely aligned with the conceptualizations put forth above), Boutet et al. [30] divided attention by presenting two overlapped images (i.e., a house and a face) with 50% transparency at study. Before each stimulus onset, participants were instructed to attend to either the house or the face. Unlike the previous studies, Boutet et al. found that DA disrupted the featural processing, while leaving holistic processing relatively intact.

Why do these studies seem to come to nearly opposite conclusions? First, Reinitz et al.’s [26] inclusion of line-drawn faces is not directly comparable to real faces. In fact, studies show that featurally constructed faces are processed in a commensurately more feature-based way [31]. This over-reliance upon featural information might have made “gluing” configurations of features (see also a definition of holistic processing more similar to Fific & Townsend [16] and System Factorial Technology framework [32]) less viable as a face processing strategy in general. Thus, this type of holistic processing would have been easier to disrupt. Second, Palmero and Rhodes’ [29] inclusion of a facial-identity DA task incorporates a distraction to holistic processing to the extent that it comes from a resource-limited, time-scale dependent pool. Studies also support that longer processing time has a limited effect on holistic processing, but instead amplifies the contributions of featural processing [31,33]. Lastly, and perhaps most compelling in light of arguments proffered by Richler et al. [6], Reinitz et al. [28] and Palmero and Rhodes [29] adopted measures (old/new configuration and face inversion, respectively) that only capture some of the four different hypothesized mechanisms underlying holistic processing, whereas Boutet et al. [30] adopted the partial composite face paradigm.

Perhaps most importantly to the current study, these previous studies instantiated DA by dividing *visual* attention. Because only a small point in the visual field may be attended to at once (for reviews see [34,35]) and many working memory models theorize separate auditory and visual resource pools [36], this method of dividing attention merely reduces the amount of time that participants may spend attending to a given area of the visual field (or time-based facial-processing biases described in [33]). In contrast, secondary auditory tasks limit attention as a general-pool resource. As such, we used an auditory DA task.

### 1.3. Strategic Attentional Allocation in Face Processing

Although disrupting attention provides some insights into its role in facial processing, we also considered it important to affect the conditions under which attention can be strategically allocated. Unlike the real world, most study/distractor/test paradigms allow participants to use relatively uniform attentional and perceptual strategies for encoding. Without awareness of the conditions that they will face during the recognition test, participants’ strategic use of attention lacks metacognitive influence. In other words, participants use a variety of brute-force intentional learning strategies because they have no idea what they will be asked to remember at test. This approach ensures that encoding affects recognition, but recognition cannot affect encoding. Likewise, when test trials come at predictable intervals, participants might use metacognition to adopt a strategy that fits those experimental conditions as opposed to a viable strategy for more variable retrieval intervals.

A more fully informed sense of the strategic allocation of resources, therefore, varies the inclusion and timing of study and test trials within a single block. To this end, we employed an experimental paradigm novel for composite face tasks: the continuous recognition task [37]. Continuous recognition paradigms intermix study and test items into a single sequence of trials, allowing participants to reach a “steady state”, wherein the decay of previously encoded information approximates the information gained by newly encoded items. Moreover, this task offers some unique advantages compared to traditional recognition memory paradigms. First, as participants receive a steady, irregular stream of study and test items, they cannot anticipate what they will next encounter in the task, thereby ensuring more active attention and minimizing rehearsal. Second, a single sequence can incorporate multiple retention intervals by manipulating the number of intervening items between study and test, permitting retention intervals to be compared within-subjects. Participants may view a test item matching a study item they have seen on the previous trial, one presented ten trials prior, or at whatever rate the researcher desires. Third, stemming from the previous point, researchers can plot the forgetting curve from short-term and long-term storage at the individual level for their stimulus classes of interest. Fourth, we contend that a continuous paradigm offers a more ecologically valid milieu of encoding and retrieval that better emulates real-life memory events (such as meeting several new people at a conference) compared to segregated study/test blocks. 

### 1.4. The Current Study

As a response to noted gaps in the literature regarding the type of DA tasks and variable retention intervals [38], the current experiment compared a non-visual DA manipulation throughout a continuous recognition task. Specifically, participants viewed an intermixed sequence of study and test trials. Study trials displayed whole faces of real people. Participants’ attention on half of the study trials was divided with an auditory attention task, whereas the other half were not. Test trials occurred at short and long term retention intervals, prompting participants to make old/new recognition decisions to the top or bottom half of the test face. In line with the complete composite paradigm, trials varied with respect to congruency (i.e., congruent, incongruent), alignment (i.e., aligned, misaligned) and face type (i.e., Irrelevant-Old, or Irrelevant-New).

We put forth the following hypotheses:

- *Processing advantage for studied face halves*. If holistic processing automatically increases sensitivity to global/configural information, then it should improve performance on previously studied face halves in context.
  - Congruent, Aligned trials containing two studied face halves (i.e., Both-Old) should have higher Hits than Congruent, Misaligned trials.
  - Congruent, Aligned trials containing two studied face halves (i.e., Both-Old) should have higher discriminability than Congruent, Aligned trials containing two unstudied face halves (i.e., Both-New).
- *Processing disadvantage for irrelevant face halves.* If holistic processing automatically reduces selective attention to face parts, then decrease performance for trials containing incongruent, irrelevant face halves.
  - Incongruent, Aligned trials containing only one novel face half should have decreased Hits (for Irrelevant-New) and increased False Alarms (for Irrelevant-Old) relative to Incongruent, Misaligned trials.
  - Incongruent, Aligned trials should have lower discriminability than Incongruent, Misaligned trials.
- *Divided Attention disadvantage for Incongruent, Aligned face halves.* If divided attention at encoding selectively impairs controlled processes, then it should impair performance requiring selective attention but not sensitivity to global/configural information.
  - Divided attention should not substantially decrease Hits for Congruent, Aligned trials containing two studied face halves (i.e., Both Old).
  - Divided attention should decrease discriminability for trials containing at least one novel face half, especially when Aligned.
- *Processing disadvantages weaken negative effects of retention interval.* Although memory declines are expected across retention intervals, failures of selective attention will exacerbate the effect by weakening memory for short term intervals, thereby reducing differences at increasing retention intervals.
  - Performance on Congruent, Aligned trials with two studied face halves (i.e., Both-Old) will show significant declines across the four levels of retention intervals, regardless of Attention.
  - Performance on Incongruent trials (both Aligned and Misaligned) will show less robust differences over retention interval.

## 2. Materials and Methods

### 2.1. Participants

Forty-four undergraduates from two US universities participated in exchange for course credit toward a research participation requirement. Power analysis with G*Power indicates this is greater than the minimum of 40 required to detect a medium (0.06 < *η^2^p* < 0.14) alignment x congruency interaction, which meta-analysis has revealed to be “moderate and robust” [18]. Participants ranged from 18 to 29 years old (*M* = 19.37, *SD* = 2.00), and consisted of 69.44% female respondents. All participants gave their informed consent for inclusion before participation. The study was conducted in accordance with the Declaration of Helsinki, and the protocol was approved by the Institutional Review Boards of the first and second author under protocols #1141837 and #2019-59.

### 2.2. Materials

All stimuli, experimental design files, and data are available at [https://osf.io/pk3x6] (accessed on 30 July 2023). Photographic facial stimuli from two sources were used in the current study: neutral (Although some facial images might be interpreted as subtly valenced, no facial expressions were reported by their original source as overtly emotional in line with traditional moods (e.g., happy, sad, angry)) faces from the Max Plank Institute for Human Development’s FACES database [39] and neutral expression faces from the first author’s personal collection of stimuli. Images depicted adult White men and women. All faces displayed a neutral expression in frontal pose with adequate lighting and crisp resolution. Each facial image was standardized by pupil location and cropped to the same oval shape to eliminate the biasing influence of external features such as hair and face shape. Final facial images were 250 × 350 RGB color JPEG images (see Figure 1). Study faces were always displayed aligned intact, with a horizontal line crossing the bridge of the nose. Test faces were either aligned or horizontally misaligned along the nose bridge by approximately 50% (see Figure 2). In addition, test faces were recombined in one of four ways: as matching top and bottom halves from a previously studied face (i.e., Both-Old), matching top and bottom halves of a completely new face (i.e., Both-New), mismatching top and bottom halves between a previously studied face and a previously new face (i.e., Irrelevant-Old, Irrelevant-New). 

Experiment programs were designed and run using E-Prime 2.0 displaying on 60 Hz monitors. A secondary task was also employed for part of the experiment where three easily discriminable tones (at low, medium, and high frequencies) were played at random during study events. Tones played through headphones. Data were analyzed using IBM SPSS 27.

### 2.3. Design

The following experiment used a 2 (Attention: Full, Divided) x 2 (Test Face Alignment: Aligned, Misaligned) x 2 (Test Face Type: Irrelevant Old, Irrelevant New) x 4 (Retention Interval: Short-term Memory (STM), Short Long Term Memory (Short LTM), Medium Long-term memory (Med LTM), and Long Long-term Memory (Long LTM)) within-subjects design. 

### 2.4. Procedure

**Figure 3:**
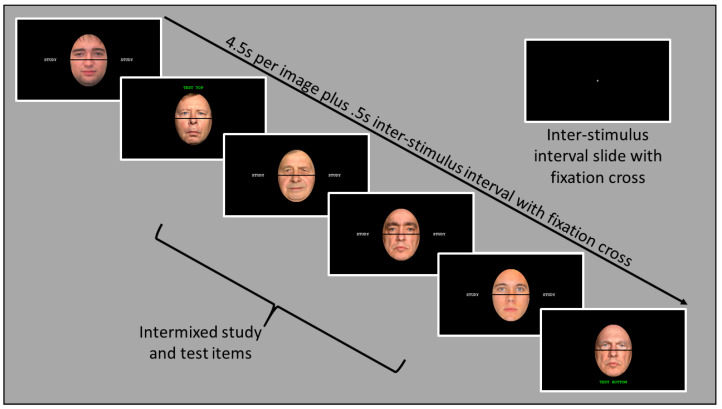
Example flow of stimuli within the continuous recognition paradigm. Test slides feature green font.

Each participant completed the experiment individually with the researchers present. After providing signed informed consent, participants listened to instructions simultaneously presented onscreen and read by the researcher, allowing for an opportunity to ask questions and clarify any aspects of the study. After a brief series of practice trials, participants engaged in the two main experimental blocks (see Figure 3 for example sequence). 

One block featured full attention (FA), whereas the other block featured divided attention (DA) brought about by a secondary task. During the DA block, participants responded to one of the three easily discriminable tones (low, medium, or high frequency) by pressing the appropriately labeled keyboard key. The order of these blocks was counterbalanced across participants.

Each block also featured an intermixed sequence of study and test slides with black backgrounds. Study slides were displayed for 4.5 s each and showed the word “STUDY” on either side of the face in white text. Test slides were displayed for 4.5 s each and varied in alignment (aligned or misaligned) and of one of four face types (1) Both-Old, where top and bottom halves were both previously studied, (2) Both-New, where top and bottom halves were entirely new, (3) Irrelevant-New, where participants were prompted to respond to a previously studied half that was paired with an unstudied half, or (4) Irrelevant-Old, where participants were prompted to respond to a previously unstudied half that was paired with a studied half. Participants were prompted to respond to either the top or bottom half of the face, with “TEST TOP” above the face or “TEST BOTTOM” below the face as appropriate in green text. Study and Test text featured different colors to facilitate participants’ ability to discriminate their instructions, and test text featured on the bottom or top of the screen to draw attention to those facial halves.

Participants answered yes/no recognition questions at each of the four retention intervals. STM was assessed when test slides followed their corresponding study slides 0, 5, or 10 s later. Short LTM intervals were assessed when test slides followed 55, 60, or 65 s later. Med LTM was assessed when test slides followed 105, 110, or 115 s after study slides. Long LTM was assessed when test slides follow 215, 220, or 225 s after study slides. In all, participants viewed 168 total slides per block with 72 study slides and 96 test slides (i.e., six per individual retention interval plus 24 distractors). These retention intervals were determined based on previous studies using such a paradigm [40,41].

After completing all study/test trials, participants answered questions about impressions about the study and the perceived difficulty of the tests. Participants also provided demographic information. The researchers then debriefed and compensated the participant. 

## 3. Results

Data were tabulated into separate hit rates, false alarm rates, and discriminability (*d’*), which were the dependent variables analyzed. Although previous literature using continuous recognition paradigms, e.g., ref. [40] has used proportion hits minus proportion false alarms (i.e., corrected recognition), *d’* is used most often in the literature examining the complete design of the composite face task. All analyses were conducted within-subjects. 

### 3.1. Hits

**Figure 4:**
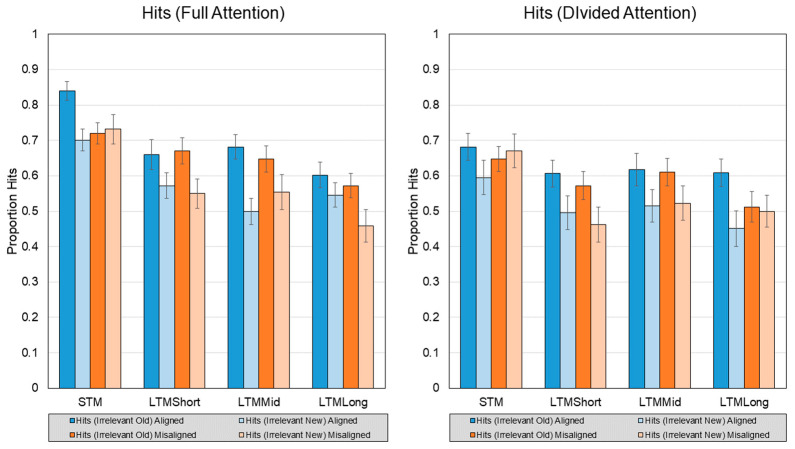
Hit rate from each attention condition, face type, and retention interval. Error bars represent mean standard error.

The first analysis (see Figure 4) examined hit rates in a 2 (Attention: Full vs. Divided) x 2 (Alignment: Aligned vs. Misaligned) x 2 (Face Type: Irrelevant Old vs. Irrelevant New) x 4 (Retention Interval: STM vs. Short LTM vs. Med LTM vs. Long LTM) repeated-measures ANOVA. Tests of sphericity revealed that the Retention Interval effect and the Alignment x Face Type x Retention Interval interaction violated the sphericity assumption. So, the Greenhouse-Geisser adjustment was used for the former and the Huynh-Feldt adjustment was used for the latter. 

Analyses revealed a main effect of Attention such that Full yielded more hits (*M* = 0.63, *SD* = 0.14) than Divided (*M* = 0.57, *SD* = 0.17), *F*(1, 43) = 8.28, *p* = 0.006, *η^2^p* = 0.161. Face Type also yielded a significant main effect, *F*(1, 43) = 51.52, *p* < 0. 001, *η^2^p* = 0.545, with Both-Old faces yielding greater (*M* = 0.64, *SD* = 0.14) than Irrelevant-New faces (*M* = 0.55, *SD* = 0.15). Retention Interval also yielded a significant main effect, *F*(2.35, 129) = 35.44, *p* < 0.001, *η^2^p* = 0.452, such that STM yielded a reliably higher hit rate than the rest (*M* = 0.70, *SD* = 0.15), Long LTM yielded the significantly lowest hit rate (*M* = 0.53, *SD* = 0.15), and Short LTM (*M* = 0.57, *SD* = 0.16) and Medium LTM (*M* = 0.58, *SD* = 0.16) did not reliably differ. 

A significant Alignment x Face Type interaction was also found, *F*(1, 43) = 11.03, *p* = 0.002, *η^2^p* = 0.204. This interaction was driven by larger effect of Face Type for Aligned faces (*η^2^p* = 0.609) over Misaligned faces (*η^2^p* = 0.282). In particular, aligned Irrelevant-New faces demonstrated greater hit rates than misaligned Both-Old faces. A significant Attention x Retention Interval interaction was uncovered, *F*(3, 129) = 2.89, *p* = 0.038, *η^2^p* = 0.063. Follow-up tests revealed that, although a main effect of Retention Interval maintained in both attention conditions, it was weaker in the Divided condition (*η^2^p* = 0.475) compared to Full (*η^2^p* = 0.608). 

No three- or four-way interactions were detected.

### 3.2. False Alarms

**Figure 5:**
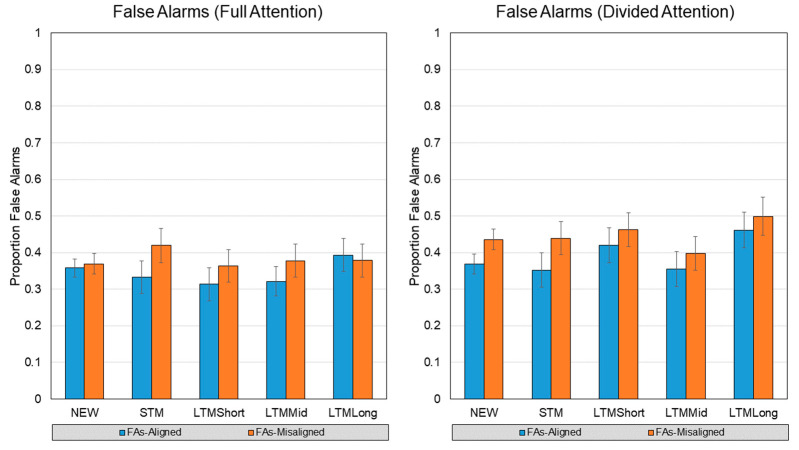
False alarm rates from each attention condition, face type, and retention interval. Error bars represent mean standard error.

The current experiment had two types of “new” test items to contribute to false alarm calculations: faces whose irrelevant halves were also new, and faces whose irrelevant halves were old. Therefore, the latter can be examined among retention intervals, but the former has no retention interval. These five cells were combined to analyze false alarms in a single 2 (Attention) x 2 (Alignment) x 5 (Retention Interval: None, STM, Short LTM, Mid LTM, Long LTM) within-subjects ANOVA. Means are displayed in Figure 5. 

This analysis found a main effect of Attention, *F*(1, 43) = 7.43, *p* = 0.002, *η^2^p* = 0.147 where DA (*M* = 0.40, *SD* = 0.14) yielded higher false alarm rate than FA (*M* = 0.36, *SD* = 0.16). We also found an effect of Alignment, *F*(1, 43) = 12.61, *p* = 0.001, *n^2^p* = 0.227, such that misaligned faces yielded higher false alarm rates (*M* = 0.41, *SD* = 0.15) than aligned faces (*M* = 0.36, *SD* = 0.14). Neither a main effect of Retention Interval nor any interactions were found. 

### 3.3. Discriminability

**Figure 6:**
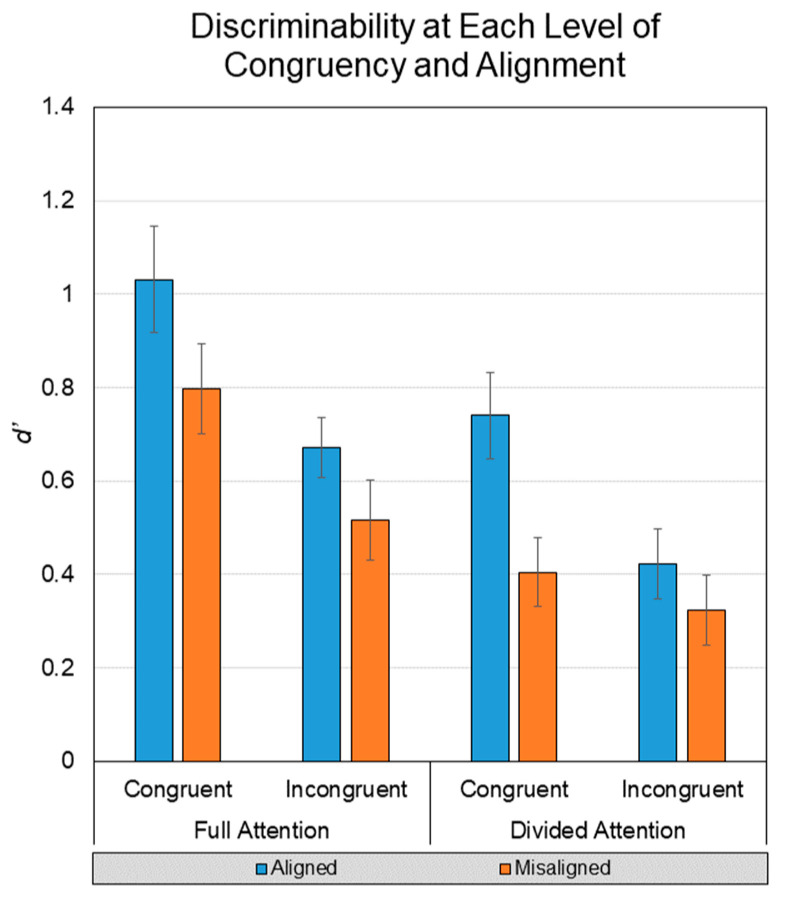
Discriminability (*d’*) calculated from all old and new items (including entirely new trials unconnected to any retention interval) in the task collapsed across retention interval. Error bars represent mean standard error. The congruency x alignment interaction provides evidence of holistic processing without the task.

The final analysis used an index of discriminability (*d’*) to examine factors previously explored for hits and false alarms and also congruence, a factor that was afforded by our use of the complete composite face paradigm. Test items are congruent if each face half have the same study status (i.e., Both-Old, Both-New), and they are incongruent if one half is studied and the other is not (i.e., Irrelevant-Old, Irrelevant-New). As described in the introduction, this permits the examination of the entire matrix of possible factorial combinations as shown in Figure 6. As with false alarms, this measure also precludes analysis of retention interval as a factor, as the entirely new test faces belong to no retention interval condition. 

As such, the final analysis included a 2 (Attention) x 2 (Alignment) x 2 (Congruence: congruent, incongruent) within-subjects ANOVA. Analyses revealed a main effect of Attention, *F*(1, 43) = 18.25, *p* < 0.001, *n^2^p* = 0.298, where FA yielded higher discriminability (*M* = 0.75, *SD* = 0.48) than DA (*M* = 0.47, *SD* = 0.36). A main effect of Alignment was also found, *F* (1, 43) = 16.69, *p* < 0.001, *n^2^p* = 0.280, where aligned faces yielded higher discriminability (*M* = 0.71, *SD* = 0.41) than misaligned faces (*M* = 0.51, *SD* = 0.39). A main effect of Congruence was also found, *F*(1, 43) = 33.69, *p* < 0.001, *n^2^p* = 0.439, where congruent faces yielded higher discriminability (*M* = 0.74, *SD* = 0.60) than incongruent faces (*M* = 0.48, *SD* = 0.46). 

The only interaction to reach significance was the Alignment x Congruency interaction, *F*(1, 43) = 5.08, *p* = 0.029, *n^2^p* = 0.106. A simple effects test of Congruence at each level of Alignment revealed that the effect of congruence was weaker for misaligned faces (*n^2^p* = 0.189) than aligned faces (*n^2^p* = 0.458). This final interaction is evidence that we replicated the composite test effect using the complete design of the composite face paradigm, at least collapsed across retention intervals. However, some readers may be curious about the evidentiary status of our failure to find the three-way interaction. Because null-hypothesis statistical testing does not permit interpretation of null findings, we used our ANOVA results for this interaction (*F*(1, 43) = 0.57) along with a null probability of 0.3 (based on the prior probability of a medium effect) to calculate *BF_01_* = 5.09, meaning that the observed data are 5.09 more likely under the null hypothesis (posterior probability = 0.68) than under the alternative two-tailed hypothesis (posterior probability = 0.32). 

## 4. Discussion

The present study examined the full model of the composite face paradigm under full and divided attention using a continuous recognition task. We found mixed support for the hypotheses presented above. Namely, although the processing advantages predicted by increased sensitivity to global/configural information were revealed, the role of dividing selective attention was less clear.

In support of the automaticity of the holistic processing advantage, we found main and interaction effects on hits and discriminability. As predicted, Both-Old face halves yielded a higher hit rate when aligned than misaligned. Although dividing attention depressed performance overall, this alignment advantage for congruent trials persisted. Similarly, retention interval produced significant declines under full attention that were weakened (but not eliminated) under divided attention, and primarily among hits within the two shortest retention intervals.

Although the advantages of holistic processing were quite clear, the disadvantages in response to failures of selective attention were not. Evidence for holistic face processing (as conceptualized by [21]) rests in the interaction between facial congruency and facial alignment—namely, error rates when a participant is tested on individual face halves are lower when those face halves are horizontally misaligned than when the faces are aligned. In this account of holistic processing, viewing an intact, aligned face triggers the perceptual system to treat the face as a unified whole. According to this premise, viewing a misaligned face makes responding to old/new recognition questions about individual halves easier, increasing accuracy. Despite the theoretical predictions, misalignment did not reduce false alarms or increase discriminability in incongruent trials. Instead, the opposite pattern emerged. For both incongruent and congruent trials, alignment benefited recognition performance. Although divided attention lowered the overall performance, an interaction supported the idea that the incongruent, aligned advantage was even more pronounced.

These latter findings run contrary to the expected patterns found elsewhere in the literature [6,26]. Importantly, these differences cannot be attributable to the relative disconnection between measures (e.g., inversion, scrambling, part/whole) and their corresponding claims about the nature of holistic processing. Two possibilities emerge, then, to shed light on our outcomes.

### 4.1. Facial Perception as a Resource-Dependent Dual Process

The first possibility is that the continuous recognition paradigm, which intermixes study and test trials, is attentionally taxing. Participants are aware that recognition trials will appear at seemingly random intervals, during which a recognition judgement must be made within 4.5 s before the screen transitions to the next trial. This knowledge, in turn, can and should inform their attentional strategies. Unlike blocked study/test designs or predictable study/test trial sequences, participants in the continuous recognition task must dynamically allocate their attentional resources to meet the demands of forthcoming recognition test trials. When the continuous recognition task is combined with an attention-demanding secondary task, perhaps relying upon any strategy that requires *overcoming* the distractions of irrelevant information infeasible. 

In response, participants might have adopted an approach that leverages the rapid, compulsory, and automatic [29] nature of the holistic processing advantage brought about by alignment. A unified whole comprised of new and old face parts should trigger weaker memories than a unified whole comprised of completely old face parts. If participants were seeking out opportunities to make quick (4.5 s) decisions that did not rely upon their limited attentional resources, that reduced signal strength could still be useful. When attention is divided, it would be a stronger asset to preserving recognition of previously studied facial information. While this strategy would not be very helpful in avoiding false alarms, it would be relatively effective in increasing hits. 

In light of our findings, perhaps other conceptualizations of holistic processing and their corresponding predictions need to be more fully considered. One promising avenue that has received quite a bit of more recent attention (e.g., [17,32,42]) concerns face perception as a product of parallel, coactive processing [16]. As summarized in [6] and subsequently expanded with real face stimuli (as opposed to featurally constructed faces, which bias towards featural processing [31]), these studies support the contention that face perception relies upon limited-capacity, serial processing. The argument not only relies upon findings from complete composite tasks, but also applies principles of system factorial technology (SFT; [43]) to face perception. 

Of interest to the current investigation, SFT is a broader conceptualization that addresses the nature (serial versus parallel) and capacity (limited, unlimited, or super capacity) of a wide variety of cognitive information-processing phenomena. It has been successfully applied to other tests of face recognition, including and beyond the complete composite design [44,45,46,47] with some success towards theoretical unification. To extend and contextualize our own findings within this literature, future work should develop paradigms that treat encoding and retrieval strategies as dynamic and responsive (such as the continuous recognition paradigm) to inform this and other theoretical claims about face processing.

### 4.2. Dynamic Contributions of Perceptual and Attentional Processing

Another promising path to interpret and integrate these findings more fully into a cohesive framework involves the integration of domain-general and domain-specific neurological contributions. Although face perception literature is replete with examples of behavior-only outcomes (as are reported here), promising new work by Chen and colleagues [22] investigated the composite face effect as a neural-behavioral phenomenon. In their study, participants completed the composite face paradigm in an MRI scanner. Results revealed independent contributions of face-selective neural regions (e.g., fusiform face area, temporal sulcus, and occipital face area) and attention-supporting neural regions (e.g., anterior insula, medial prefrontal cortex, inferior parietal lobe). Further, patterns differed between results from the composite face paradigm and other facial processing tasks (i.e., Eriksen Flanker Task [48]). The authors, therefore, argued that the composite face effect relies on both networks. Even if participants do not produce an overt motor response (e.g., yes/no recognition), covert decision-making strategies (e.g., attentional allocation) support performance.

Again, we argue that the continuous recognition task and other paradigms that require dynamic, strategic attention allocation might be useful in adjudicating between the many conceptualizations of holistic processing. To the extent that holistic processing is neither unidimensional nor static, anatomical/functional and behavioral findings can more strongly test the contributions of featural and holistic information in response to task demands.

## Data Availability

All data and materials can be found at OSF [https://osf.io/pk3x6] (accessed on 30 July 2023).
